# Implementation of a fully remote randomized clinical trial with cardiac monitoring

**DOI:** 10.1038/s43856-021-00052-w

**Published:** 2021-12-20

**Authors:** Jacob J. Mayfield, Neal A. Chatterjee, Peter A. Noseworthy, Jeanne E. Poole, Michael J. Ackerman, Jenell Stewart, Patricia J. Kissinger, John Dwyer, Sybil Hosek, Temitope Oyedele, Michael K. Paasche-Orlow, Kristopher Paolino, Paul A. Friedman, Chloe Waters, Jessica Moreno, Hannah Leingang, Kate B. Heller, Susan A. Morrison, Meighan L. Krows, Ruanne V. Barnabas, Jared Baeten, Christine Johnston, COVID-19 Early Treatment Team, Medhavi Bole, Alyssa Braun, Helen Y. Chu, Mark Drummond, Kirsten Hauge, Madelaine Humphreys, Abir Hussein, Christine Johnston, Steve Kuntz, Anya Mathur, Lindsey McClellan, Jessica Moreno, Thepthara Pholsena, Matthew Seymour, Helen Stankiewicz-Karita, Jenell Stewart, Jina Taub, Zoe Thuesmunn, Ethan Valinetz, Dana Varon, Anna Wald, Brian Wood, Maianna Dematteis, Katie Wicklander, Rebecca Letterer, Jeanne Poole, Arun R. Sridhar, Jeff Purcell, Mary Kirk, Chloe D. Waters, Jared M. Baeten, Ruanne V. Barnabas, Jennifer Baugh, Clare E. Brown, Connie Celum, Daphne Hamilton, Harald S. Haugen, Rachel Johnson, Jack Knauer, Caroline H. Liou, Susan Morrison, Justice Quame-Amaglo, Azaad, Randy Stalter, Jenell Stewart, Katherine Thomas, Vianey Vazquez, Grant E. Young, Yasaman Zia, Azaad Zimmermann, Meei-Li Huang, Alexander L. Greninger, Keith R. Jerome, Mark H. Wener, Deborah J. Brown, Nathaniel Davenport, Omar Gambito, Luisa Arroyave, Agata Bereznicka, Jonathan Berz, Pablo Buitron, Michael Camuso, Leticia Cardoso, Ricardo Cruz, Julien Dedier, Husam Dennaoui, Anna Goldman, Lori Henault, Terrell Johnson, Sarah Koberna, Carlie Martinez, Erin Martinez, Crystal Ng, Michael Paasche-Orlow, Margot Rogers, Kathleen Salerno, Carl Streed, Ve Truong, Nisha Verma, Katherine Waite, Steven Zalewski, Elizabeth R. Brown, Tracy Q. Dong, Joshua Schiffer, Chris Balthazar, Kelly Bojan, Hamid Bouiri, Marisol Consignado, Kortez Davis, Sadhana Dharmapuri, Mireya Gonzalez, Sybil Hosek, Rachel Jackson, Meenakshi Malhotra, Antionette McFadden-Smith, Raymond McPherson, Ryan Muench, Ixchell Oritz-Estes, Temitope Oyedele, Dorothy Rego, Zoe Ellen Sanders, Alisa Seo-Lee, Karen Simpson, Michael J. Ackerman, Zachi I. Attia, Peter A. Noseworthy, Stefanie E. Bendik, Anna Bershteyn, Robert A. Pitts, Peter Greco, Michelle Klick, Kristopher M. Paolino, Mueenah Anibaba, Evan Atkinson, Mary Beth Campbell, Gerard Gomes, Jacob Hall, John Huntwork, Margaret Huntwork, Patricia Kissinger, Heather Larkin, Cedrick Ntambwe, Florice Numbi, Michelle Paloomares, Norine Schmidt, Hamada Rady, Maria Ribando, Daniel Triggs, Neha Upadhyay, Crystal Zheng, Arun R. Sridhar

**Affiliations:** 1 Division of Cardiology, University of Washington, Seattle, WA USA; 2 Division of Heart Rhythm ServicesDepartment of Cardiovascular Medicine, Mayo Clinic, Rochester, MN USA; 3 Division of Allergy and Infectious Diseases, University of Washington, Seattle, WA USA; 4 Department of Global Health, University of Washington, Seattle, WA USA; 5 Tulane University, New Orleans, LA USA; 6 Rush University Medical Center, Chicago, IL USA; 7 John H. Stroger Jr., Hospital of Cook County, Chicago, IL USA; 8 Boston University School of Medicine, Boston Medical Center, Boston, MA USA; 9 State University of New York Upstate Medical University, Syracuse, NY USA; 10 Division of Allergy and Infectious Diseases, Department of Medicine, University of Washington, Seattle, WA USA; 11 Institute of Translational Health Sciences, University of Washington, Seattle, WA USA; 12 Division of Cardiology, Department of Medicine, University of Washington, Seattle, WA USA; 13 Department of Pharmacy, University of Washington, Seattle, WA USA; 14 Department of Medicine, International Training and Education Center for Health, University of Washington, Seattle, WA USA; 15 Department of Laboratory Medicine, University of Washington, Seattle, WA USA; 16 Virginia Mason Memorial Hospital, Yakima, WA USA; 17 Boston Medical Center, Boston, MA USA; 18 Fred Hutchinson Cancer Research Center, Seattle, WA USA; 19 Mayo Clinic, Rochester, MN USA; 20 NYU Grossman School of Medicine, New York, NY USA; 21 SUNY Upstate Medical University, Syracuse, NY USA

**Keywords:** Randomized controlled trials, Cardiology

## Abstract

**Background:**

The coronavirus disease 2019 (COVID-19) pandemic has challenged researchers performing clinical trials to develop innovative approaches to mitigate infectious risk while maintaining rigorous safety monitoring.

**Methods:**

In this report we describe the implementation of a novel exclusively remote randomized clinical trial (ClinicalTrials.gov NCT04354428) of hydroxychloroquine and azithromycin for the treatment of the SARS-CoV-2–mediated COVID-19 disease which included cardiovascular safety monitoring. All study activities were conducted remotely. Self-collected vital signs (temperature, respiratory rate, heart rate, and oxygen saturation) and electrocardiographic (ECG) measurements were transmitted digitally to investigators while mid-nasal swabs for SARS-CoV-2 testing were shipped. ECG collection relied on a consumer device (KardiaMobile 6L, AliveCor Inc.) that recorded and transmitted six-lead ECGs via participants’ internet-enabled devices to a central core laboratory, which measured and reported QTc intervals that were then used to monitor safety.

**Results:**

Two hundred and thirty-one participants uploaded 3245 ECGs. Mean daily adherence to the ECG protocol was 85.2% and was similar to the survey and mid-nasal swab elements of the study. Adherence rates did not differ by age or sex assigned at birth and were high across all reported race and ethnicities. QTc prolongation meeting criteria for an adverse event occurred in 28 (12.1%) participants, with 2 occurring in the placebo group, 19 in the hydroxychloroquine group, and 7 in the hydroxychloroquine + azithromycin group.

**Conclusions:**

Our report demonstrates that digital health technologies can be leveraged to conduct rigorous, safe, and entirely remote clinical trials.

## Plain language summary

Clinical trials have traditionally been carried out at biomedical research institutions, such that participants travel to these hubs for treatment and monitoring. This report describes an entirely remote clinical trial with the use of digital technologies to perform data collection and cardiac monitoring, in which participants self-collected vital signs and electrocardiogram (ECG), using the internet to transmit this information to trial organizers. This trial tested two medications, hydroxychloroquine and azithromycin, for the treatment of COVID-19. These compounds can result in serious cardiac side effects related to abnormal electrical conduction in the heart, and thus require active cardiac monitoring with an ECG. We show that remote cardiac monitoring is feasible using digital technologies. This paradigm may help improve clinical trial accessibility, promote participant diversity, improve trial efficiency, and reduce exposure risk of participants and staff to infectious diseases.

## Abstract (web summary)

Mayfield et al. describe the implementation and feasibility of an exclusively remote randomized clinical trial of hydroxychloroquine and azithromycin for the treatment of COVID-19. The trial included monitoring of cardiovascular safety which was successfully done using digital technologies.

## Introduction

Coronavirus disease 2019 (COVID-19), caused by infection with the severe acute respiratory syndrome coronavirus 2 (SARS-CoV-2), has spread to more than 194 million people and caused more than 4 million deaths globally as of July 2021^1^. In the context of a critical need to stem the morbidity and mortality of the SARS-CoV-2 pandemic, the development and testing of therapeutics has proceeded at an unprecedented pace^2–5^. Hydroxychloroquine and azithromycin, two widely available drugs, were of intense interest at the outset of the pandemic given their potential efficacy against SARS-CoV-2 based on in vitro and early observational data^6–8^. Therefore, we designed a randomized, placebo-equivalent trial to test two therapeutic regimens—hydroxychloroquine and hydroxychloroquine combined with azithromycin—for efficacy in reducing disease progression in ambulatory patients with COVID-19. In light of concerns regarding potential cardiovascular toxicities related to hydroxychloroquine- and azithromycin-induced QT prolongation, this trial was designed to rigorously evaluate the cardiovascular safety of these regimens^9–14^. Although hydroxychloroquine has been shown to be ineffective for the treatment of COVID-19^15–17^, our experience with digital cardiac safety monitoring provides important proof of principle for a technology that can be applied broadly to facilitate more pragmatic, “site-less,” and increasingly digital clinical trials of the future. Such remote trials may significantly improve access for patients who live in remote underserved places, work, have children, and/or lack transportation, which are common barriers to trial participation, and thus may help democratize clinical trials.

In this study, we utilized digital technologies that facilitated collaboration among scientists, implemented internet-based recruitment and enrollment, and leveraged rapid computer-assisted data analysis. Here, we describe our experience in the design and execution of this fully remote clinical trial, using digital technologies and infrastructure to provide real-time ascertainment of cardiovascular safety and risk in the evaluation of an investigational drug regimen for COVID-19.

There are three key elements of a digital clinical trial: recruitment and retention, patient-reported and -collected health data, and digital analytics^18^. Digital recruitment and analytics techniques are already in widespread use, as evidenced by several recent remote COVID-19 trials^19–23^, but the incorporation of real-time digital health data collection in clinical trials has been limited.

We conducted a trial that incorporated real-time digital health data collection. The cardiovascular safety concerns inherent to hydroxychloroquine and azithromycin necessitated monitoring of the QT interval using the electrocardiogram (ECG). We hypothesized that patients could check their own vital signs and self-administer nasal swabs, a technique that is comparable in sensitivity to healthcare workers who were administered nasopharyngeal swabs^24^, as well as remotely collect ECG tracings to monitor QT intervals. Previous trials of atrial fibrillation have relied on remote rhythm monitoring with Holter monitors or wearable extended recording devices to assess outcomes^25,26^, but these modalities are limited in that they are adjudicated post hoc after being physically mailed to a processing facility. When QT interval monitoring is a relevant concern, active surveillance is typically required due to potential progression to torsades de pointes with continued dosing of the inciting agent after QT prolongation has developed^27^.

This dictated the rapid development of a system of remote ECG collection, analysis, and reporting that was easy for participants to self-administer and would provide accurate, timely, and clinically meaningful data. While there are numerous consumer ECG devices on the market, the fact that multilead ECG tracings are necessary to make an accurate assessment of the QT interval^28,29^ favored the selection of a device capable of simultaneous six-lead recording. We utilized the KardiaMobile 6L, the first device cleared by the US Food and Drug Administration for use in monitoring QT interval^30^.

## Methods

### Design and oversight

In this multicenter, double-blind placebo-controlled trial, participants were randomized into three arms in a 1:1:1 fashion to hydroxychloroquine + placebo (folic acid), hydroxychloroquine + azithromycin, and a placebo-equivalent control (ascorbic acid + folic acid). The complete methods have been previously published^17^. The study was registered at ClinicalTrials.gov (NCT04354428).

**Fig. 1:**
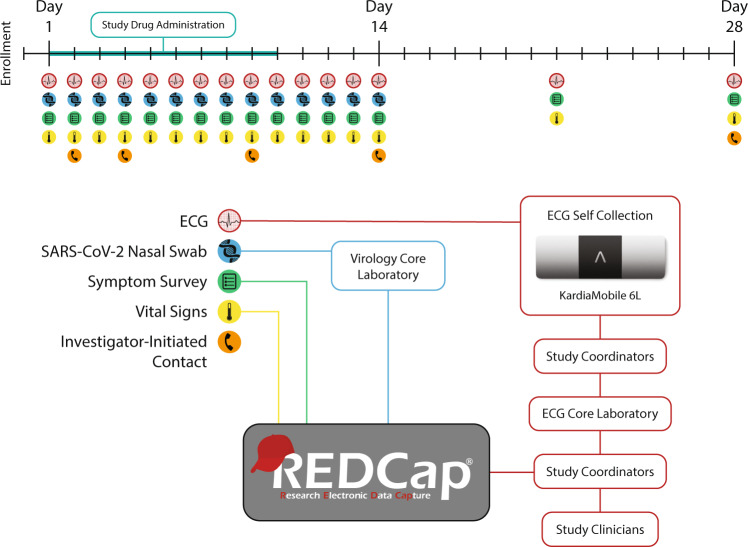
Timeline of participant study drug administration and data collection with schematic demonstrating data processing and storage. Visual representation of the study protocol. Study drugs were taken on protocol days 1–10. ECGs, mid-nasal viral swabs, symptom surveys, and vital signs were self-collected on protocol days 1–14, while only ECGs, symptom surveys, and vital signs were obtained on days 21 and 28. On days 2, 4, 9, 14, and 28, planned investigator-initiated contact was undertaken to collect subjective data and encourage adherence. Vital signs were collected twice daily. ECGs were uploaded to the secure web portal where study coordinators pushed them to the core ECG laboratory and then recorded the returned interpretation, as described in the “Methods” section. Viral swabs were sent to the core virology laboratory. All data were warehoused in REDCap^33^.

The trial was conducted entirely remotely (Fig. 1), from enrollment through data collection and follow-up, with the first enrollment occurring on 16 April 2021 and the last enrollment date being 28 July 2021. Five US institutions enrolled participants. Written informed consent was obtained from all participants. The study was approved by the Western Institutional Review Board with reliance agreements from the collaborating institutions. An external and independent data and safety monitoring board provided oversight.

### Inclusion and exclusion criteria

Individuals between the ages of 18 and 80 who tested positive for SARS-CoV-2 via polymerase chain reaction (PCR) assay within the prior 72 h were eligible for inclusion provided they possessed access to the internet for participation in video conference visits and to complete study data. Exclusion criteria included a personal or family history of long QT syndrome, concurrent use of other QT interval prolonging drugs (e.g., ondansetron, citalopram), and heart failure with New York Heart Association Class II or worse symptoms (full inclusion/exclusion criteria described elsewhere)^17^.

### Recruitment and data collection

**Fig. 2:**
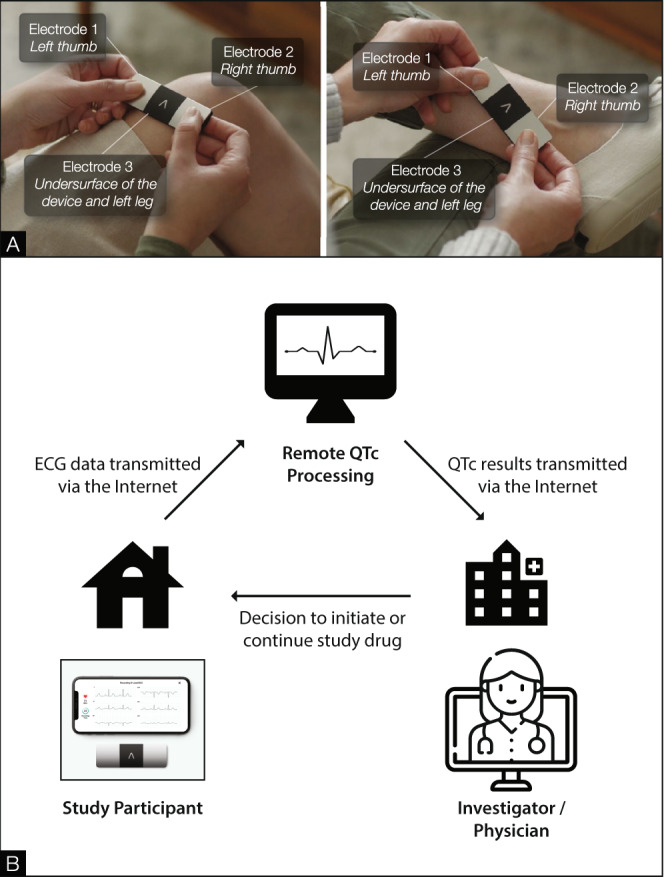
Electrocardiogram acquisition and workflow. Six-lead electrocardiogram acquisition by the patient using the AliveCor® KardiaMobile 6L device (**A**). Note: rotating the device 180° results in limb lead reversal. Implementation of remote QTc monitoring (**B**). ECG is collected by the participant, uploaded to a secure web portal, and transmitted by study coordinators for adjudication at a core laboratory. QTc results are transmitted to study coordinators for safety ascertainment by study clinicians.

**Fig. 3:**
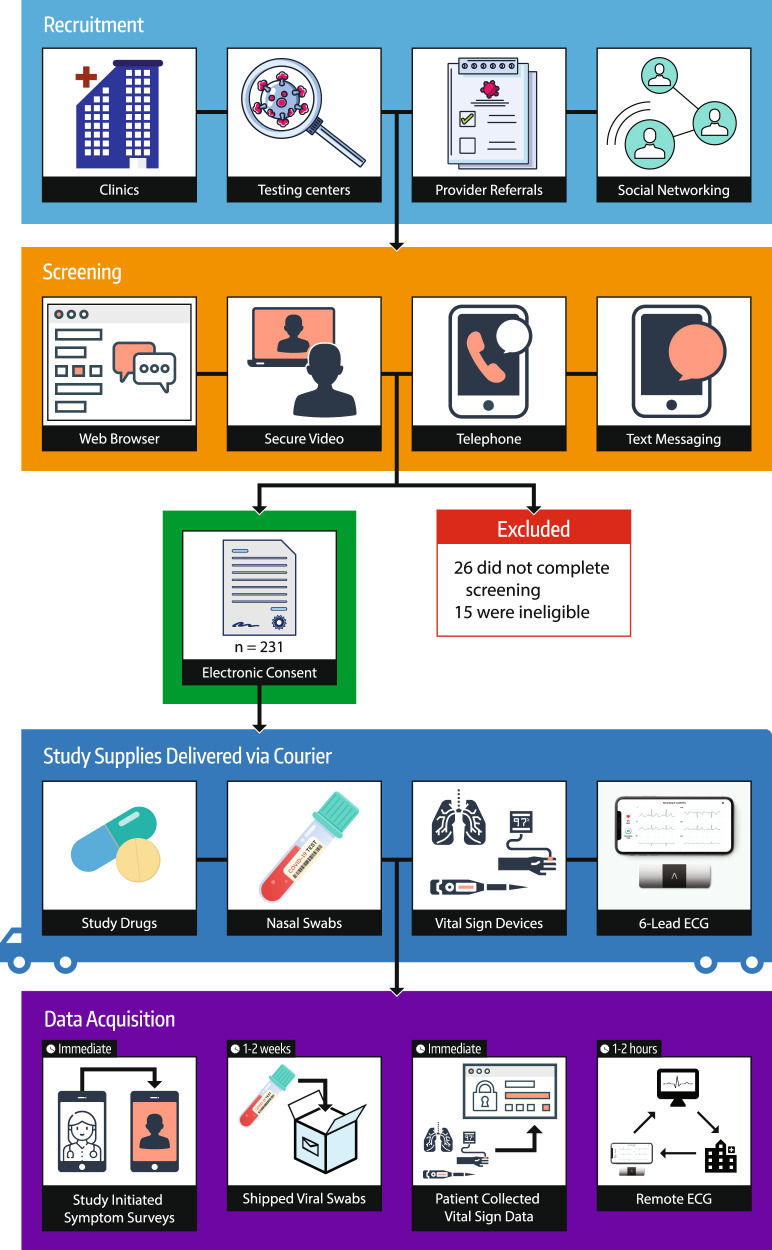
Graphical overview of recruitment, screening, logistics, and data acquisition. A multipronged recruitment effort was utilized, and screening leveraged secure digital health technologies.

Nationwide social media advertising was employed for recruitment. Participants were screened via the web interface, secure video conference, telephone, or text message. Electronic informed consent was obtained in English or Spanish through a secure video conference. A randomization sequence was utilized to assign households 1:1:1 to the three arms of the trial, stratified by high- or low-risk cohort, as previously described^17^. A courier delivered study drugs, a self-monitoring kit including a six-lead ECG monitor (Fig. 2; KardiaMobile 6L, AliveCor®, Mountain View, CA), an oxygen saturation (SpO_2_) monitor (Vive Precision), and an oral thermometer (Adtemp IV), as well as mid-nasal swabs for SARS-CoV-2 RT-PCR testing^31^ to each participant. On days 1–14 and on days 21 and 28, participants self-collected mid-nasal PCR swabs, obtained vital signs including temperature, respiratory rate, heart rate, and oxygen saturation, completed daily symptom surveys (modified Flu-PRO^32^), and uploaded an ECG (Fig. 1). Participants packaged and returned mid-nasal swabs using provided prepaid shipping labels and containers. Data were collected and curated using REDCap electronic data capture tools hosted at the University of Washington^33^. The overall workflow is described in Fig. 3.

### ECG acquisition and analysis

Participants were provided with written and verbal instructions regarding the use of the KardiaMobile 6L device, including the collection of ECGs and submission of the data to the secure internet interface. To use the device, participants downloaded the Kardia application on their personal devices (smartphone or tablet). If a participant’s device was incompatible^34^, a smartphone was provided by the study. For Spanish-speaking participants, the default language of the Kardia™ application was switched to Spanish. Each participant was given instructions by study staff on the day of kit delivery via secure video conference. During this visit, a study coordinator helped participants download the application and set up an account, and link the device to the study. The coordinator demonstrated this process via video conference, requested the participant replicate the steps, and verified the collection of an ECG from the participant in real time. This also served as the baseline ECG.

To collect ECG tracings, participants were instructed to open the Kardia™ application on their mobile device, select “record EKG,” and then hold the KardiaMobile 6L device with their thumbs on the anterior silver spaces and the back of the device resting on their bare left knee or ankle for at least 30 s (Fig. 2). The Kardia™ application automatically identified poor quality or noisy tracings at the end-user level, prompting participants to repeat acquisition. Participants were identified within the portal by their study ID, and each site was able to track and monitor their own participants in order to contact those with adverse events (AEs) necessitating study drug discontinuation.

Standard ECG readings from KardiaMobile provide a rhythm strip but not QT intervals. Therefore, we created a real-time system to adjudicate QT intervals at a central facility with results returned to sites. The study facing secure Kardia web interface was queried several times daily and ECG submissions were tracked. The QT interval reading was requested on the first ECG collected each day (Fig. 2B). This action resulted in the transmission of deidentified ECGs to a central core laboratory (Mayo Clinic Heart Rhythm and Physiologic Monitoring Laboratory, Mayo Clinic, Rochester, MN) where the QT interval was measured by certified rhythm analysis technicians. Fiducial points were tagged on the tracing using an automated algorithm (QRS onset, T wave offset, etc.) and the RR interval, QT interval, and corrected QT interval (QTc) (Bazett and Fredericia corrections) were calculated. The ECG technologist then manually adjudicated these points and adjusted the fiducial points and confirmed the measurements. The six-lead tracings were displayed in a “QT dashboard” user interface that was developed for the purpose of this study. Each participant’s heart rate QTc values were returned to the investigator within 1 h of uploading in order to be available at the point of care. If the QT interval was not able to be calculated, the result was read as “0” and another ECG was requested from the participant.

### Qualitative data collection

For the purpose of this paper, the authors (J.M., A.S.) conducted interviews and corresponded extensively with study staff regarding qualitative experience with this paradigm. We specifically focused on practical challenges encountered at each step of the trial and the ad hoc solutions to overcome them. The results of this process are reported in the experience section of this manuscript and inform our discussion.

### Outcomes

The primary outcomes (development of lower respiratory tract infection, hospitalization, or death attributable to COVID-19) and secondary outcomes (time to cessation of viral shedding and time to resolution of COVID-19 symptoms) of the study are presented separately^17^. The data described in this paper include demographics, protocol adherence, QT prolongation-related AE), and qualitative experience derived from interviews with investigators and study coordinators.

ECGs were monitored for development of QT prolongation, defined as a QTc value ≥500 ms or an increase of 60 ms or more above baseline, either of which constituted a grade 3 AE and necessitated study drug hold while obtaining a prompt follow-up ECG assessment. Participants were contacted to collect an additional ECG. If the follow-up ECG confirmed a prolonged QT interval, the study medication was permanently discontinued.

### Reporting summary

Further information on research design is available in the Nature Research Reporting Summary linked to this article.

## Results

Two hundred and seventy-one potential participants were screened for inclusion. Twenty-six did not complete the screening procedures and an additional 15 were ineligible. No participants were ineligible due to the lack of a compatible internet-enabled device. A total of 231 participants were included from 205 households, of which 218 initiated study medication and transmitted ECG data (Table 1). The median age of participants was 37 (range 18–78) and 19 (8.7%) were 60 or older. Participants identifying as Hispanic or Latinx made up 29.8%. English was the preferred language of 90.8 and 9.1% preferred Spanish. Compared to the average US population, this trial was more diverse. Those self-identifying as white make up a smaller share in our cohort, 51.4 vs 76.3%, and fewer participants were 60 years of age or older (8.7 vs 21.5%)^35^. Once enrolled, no participant was terminated for technical challenges relating to ECG acquisition.

**Table 1:** Characteristics of per-participant ECG collection.

Statistic	Value
Total patients	219
Total ECGs	3245
Median ECGs per patient	17
Mean ECGs per patient	15.9
Standard deviation	3.9
Range of ECGs per patient	1–32

**Fig. 4:**
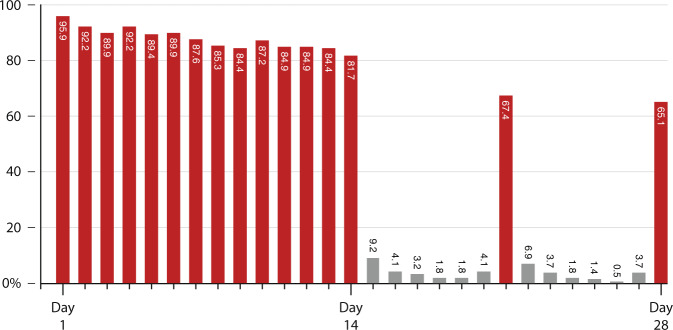
Percent of participants who submitted an ECG per protocol day. Red bars represent days that were specified for ECG submission in the protocol, while gray bars represent days when ECG upload was not required.

**Fig. 5:**
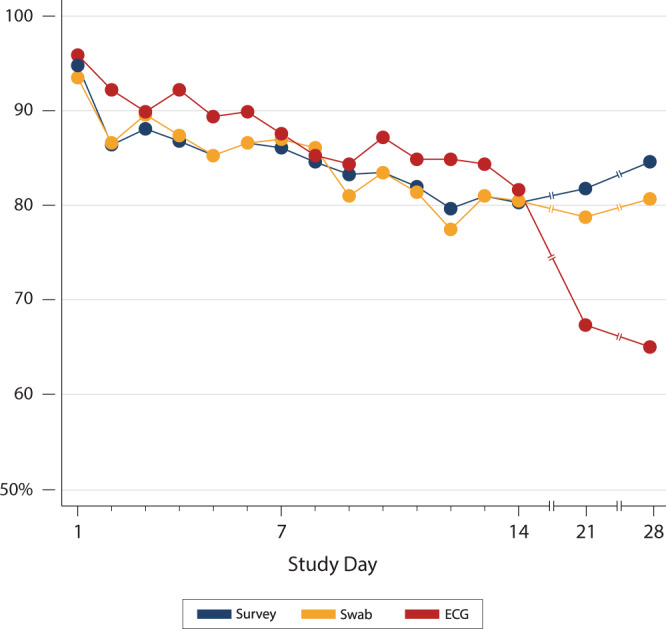
Comparative daily percent adherence to survey completion, mid-nasal swab submission, and ECG upload by protocol day. ECG protocol adherence was noted to reduce to <70% after the study drugs were discontinued after day 14.

A total of 3245 ECGs were uploaded and adjudicated. Because some participants submitted more or fewer recordings than requested, the number of ECGs per participant varied. The median number of uploaded ECGs per participant was 17 (range 0–32, standard deviation 3.9). Mean daily adherence to the ECG testing protocol throughout the study period was 85.2% (Fig. 4 and Supplementary Data File 1) and was similar to adherence to the survey and mid-nasal swab elements of the study (Fig. 5). ECG submission was high during days 1–14 (87.9%), but declined on days 21 and 28 (66.3%). ECG protocol adherence rates did not differ by age or sex assigned at birth and were high across all reported races and ethnicities (Table 2).

**Table 2:** Average ECG protocol adherence (proportion of expected study days with ECG) stratified by demographic characteristics.

ECG protocol adherence by demographic characteristics		
	No. (%)	Mean adherence (±SD)
Age		
≥60	19 (8.7)	92.1% (22.7)
<60	199 (91.3)	84.0% (15.3)
Sex assigned at birth		
Female	121 (55.5)	86.3% (20.6)
Male	97 (44.5)	82.5% (24.2)
Race		
American Indian or Alaskan Native	38 (17.4)	86.2% (20.7)
Asian	10 (4.6)	88.1% (20.5)
Native Hawaiian or Pacific Islander	3 (1.4)	70.8% (20.1)
Black	23 (10.6)	77.7% (27.6)
White	112 (51.4)	81.2% (18.2)
Other	29 (13.3)	73.3% (30.5)
Prefer not to say	3 (1.4)	98% (3.6)
Ethnicity		
Not Hispanic/Latinx	153 (70.2)	87.6% (18.0)
Hispanic/Latinx	65 (29.8)	77.7% (29.1)
Preferred language		
English	198 (90.8)	85.8% (20.9)
Spanish	20 (9.2)	73.1% (31.4)

*±SD* standard deviation.

QTc prolongation meeting criteria for an AE occurred in 28 (12.1%) participants, with 2 occurring in the placebo group, 19 in the hydroxychloroquine group, and 7 in the hydroxychloroquine + azithromycin group (Table 3). The majority of AEs were increased from baseline QTc of ≥60 ms—only two participants (0.9% of total enrolled), both of whom were in the hydroxychloroquine group, experienced prolongation of the QTc to greater >500 ms resulting in discontinuation of the study drug (Table 1).

**Table 3:** Patient-level adverse events related to QTc prolongation.

Randomized arm	ECG QTc ≥60 ms change from baseline^a,b^	ECG QTc >500 ms^a,b^	Total QTc-related adverse events^b^	Total participants
Ascorbic acid + folic acid	2 (2.5)	0 (0)	2 (2.5)	80
Hydroxychloroquine + folic acid	19 (29.2)	2 (3.1)	19 (29.2)	65
Hydroxychloroquine + azithromycin	7 (9.5)	0 (0)	7 (9.5)	74
Total	28 (12.7)	2 (0.9)	28 (12.8)	219

^a^Columns are not mutually exclusive (all ECG QTc >500 ms were also ≥60 ms change from baseline).

^b^Values are the number of participants followed by the percent of total patients in the row within parentheses.

Because this was the first study to employ this paradigm, study coordinators developed ad hoc techniques to optimize enrollment and data collection processes. The initial secure video conference contact with participants was felt to be key to the successful setup of the remote ECG monitoring system. Study coordinators reported spending approximately 1 h reviewing contents of study supplies at the time of enrollment with each participant. This strategy was felt to be particularly effective when paired with proactively sending the setup instructions for the Kardia™ application to participants via text message prior to the enrollment visit. In addition, the inclusion of study-specific screenshots was useful. A specific tracking system was not employed to address trouble-shooting needs, and thus we do not have the quantitative data on such issues. Only four (0.18%) participants did not have phones that were compatible with the ECG acquisition application. We sent these participants smartphones to use during the study with the Kardia™ application installed on the device.

### Patient recruitment

Informed consent process and randomization part were feasible both for English and Spanish-speaking patients. No major technical challenges were observed with this process.

### Equipment delivery

Equipment was delivered to the doorstep of individual participants through hired local couriers or an overnight delivery service with the goal of delivering it within 24 h of participant recruitment and randomization. This process was executed with no major challenges.

### Patient education and data collection

Participants reported to staff that thermometer and oxygen saturation monitors were easy to use and teaching the use of mid-nasal swabs was reported by study coordinators to be straightforward. Teaching the use of an ECG monitor was noted to be a more challenging and time-intensive step. Setup of the ECG device took between 10 min and 2 h depending on the baseline digital fluency of the participant. Nineteen participants (8.7%) were 60 years of age or older—study coordinators reported success in teaching the installation and the ECG acquisition process in this group. Occasionally, participants required family member support to help set up the Kardia application. While discrete numeric data are not available, a minority of participants experienced challenges with ECG acquisition during the first 2 days. Errors included the reversal of the right- and left-sided electrodes and motion artifact, which led to difficulty in QTc measurement. Subsequent telephonic teaching sessions led to the correction of these errors. We experienced, on two occasions, a potentially serious issue in which two participants in the same household attempted to pair their smartphones with their respective KardiaMobile 6L devices at the same time, but instead paired with the device assigned to the other member of the household. This theoretically could have resulted in recording ECG data for the incorrect participant if it had not been identified by study staff during telephonic follow-up interviews.

### Data interpretation and real-time analytics

Adjudication of QTc relied on partnership with a core ECG laboratory (Mayo Clinic). The major challenge to performing reproducible measurements was poor signal quality. The use of the median beat allowed the display of an interpretable signal in most cases (with minimal noise). In ambiguous cases, the technician could find an interval that was relatively free of artifact in order to make a useable measurement. Study coordinators found the process of transmitting the ECG to the core laboratory and retrieving the QTc interpretations to be user friendly.

The time between requesting review and receiving a QTc result was generally 1 h or less. There were, however, two episodes when a data transfer interruption between the core laboratory and AliveCor® servers resulted in an approximately 24-h delay, after which a backlog of QTc interpretations was received. Each uploaded ECG resulted in approximately 5 min of work for coordinators between requesting adjudication, recording the result, and informing study clinicians if necessary.

## Discussion

The COVID-19 pandemic has spurred innovation across health care. Telehealth has been rapidly scaled up to provide telephone and secure video conference services to replace care normally administered in person^36^. It is likely that many of these innovations will remain in place after the pandemic is contained, as they expand access to healthcare and reduce cost^37–39^. The same technologies that have been leveraged to respond to the pandemic have the potential to durably reshape the execution and scope of clinical trials including improving efficiency and enhancing the diversity and inclusivity of participants, but at the risk of disadvantaging those with lower levels of technological literacy or access. From the perspective of the participant, remote clinical trial designs remove transportation and scheduling barriers and could be a method of reducing participation bias in clinical trials. Our experience shows that it is feasible to conduct rigorous, yet entirely remote, decentralized clinical trials involving robust QT monitoring by leveraging digital health technologies.

Cardiovascular monitoring was critical in this trial of QT-prolonging medications and is a cornerstone of safety assessment in many clinical trials. Numerous medications are known to prolong the QT interval to varying degrees, resulting in increased risk for torsades de pointes^14^. The United States Food and Drug Administration requires that all new medications must be rigorously evaluated for potential QT interval prolongation^40^. Standard 12-lead ECG monitoring for QT prolongation incurs significant cost and there is often a delay between obtaining ECGs and interpreting results^41^. At scale, beyond the safety implications of having more rapid results, remote monitoring similar to that which we describe may improve cost-effectiveness of future clinical trials, but further study is needed. In addition, this approach may be preferable to patients as they need not wear a device and may appreciate the information provided to them in real time. Furthermore, the use of artificial intelligence to determine the QT interval was recently shown to be feasible, and may further accelerate turnaround time by focusing human attention on over-reads, minimizing time requirements, and permitting scalability^29^.

Remote digital clinical trials have the potential to expand participation to populations not traditionally included in studies including those who live far from academic health centers, people with disabilities that limit their mobility, and potentially people who live in areas with limited access to clinical research. They may also be more available to participants who work, have children, lack transportation, etc. This study shows that with proper instruction, complex technological processes can be taught to participants remotely. However, care must be taken to provide adequate staff support to avoid exclusion or unintentional disadvantaging of populations with lower levels of technological access and literacy (the “digital divide”)^42^. In a postpandemic world, the ideal balance of digital and traditional clinical trial mechanics will depend upon the characteristics of individual future trials.

The current study demonstrates that the QT interval can be efficiently measured and verified within a remote clinical trial paradigm. The drugs evaluated in our study were associated with a relatively low event rate. Further study may be warranted prior to the use of this paradigm to test drugs with very high rates of QT related adverse events.

In addition to applications in clinical trials, remote digital ECG monitoring may be a means to avoid hospitalization in certain low-risk patients being started on QT-prolonging regimens such as dofetilide or sotalol that have traditionally required hospital admission for 12-lead ECG monitoring^43,44^. Further, many commonly used medications, such as fluoroquinolones, macrolides, and methadone prolong the QT interval^45^. The ability to monitor QTc remotely may provide solutions to safely prescribe a diverse array of medications^46^. The role of remote ECG monitoring in these populations warrants further investigation.

There are several limitations to this paradigm. Importantly, real-time ECG transmission and analysis requires that participants have a compatible internet-connected device. Further, ECG acquisition and transmission require familiarity with the basic use of digital technologies. Although we did not note that that this was a significant barrier in our trial population, due to the recruitment strategy which involved internet and social media campaign, our participants could be a self-selected population. Although sample size and the post hoc nature of this report limit statistical evaluation, ECG adherence appears to have been lower in primarily Spanish-speaking participants.

In addition, given that QT interval prolongation can result in serious clinical consequences, it is critical that study staff be able to reliably contact participants to instruct them to discontinue the study drug, if necessary. Daily adherence to ECG collection was greater than 80% during study drug administration and performance was comparable to the other elements of the study. A system of automated daily prompts to improve protocol adherence may be useful in future studies. This was demonstrated to be an achievable paradigm in our trial, but our SARS-COV-2-positive participants were likely easier to reach due to quarantine restrictions. Finally, while we utilized a specific commercial device, the development of interoperable, open-source software and hardware to collect, analyze, and store remote ECG data would expand the potential scope of using digital technologies in a similar setting.

The novel design of the remote trial generated practical difficulties that were solved ad hoc and not quantitatively tracked. The authors have compiled this information through coordinator and investigator interviews and have presented these data qualitatively where quantitative information was impossible to derive.

In this report, we demonstrate that digital health technologies can be leveraged to execute entirely remote clinical trials with cardiac monitoring, enabling research to safely proceed in times of public health crisis.

## Supplementary information

Description of Additional Supplementary Files

Supplementary Data 1

Reporting Summary

## Data Availability

Source data are provided in Supplementary Data 1. The remaining data are available from the corresponding author upon reasonable request.

## References

[1] World Health Organization. *Coronavirus disease (COVID-19) Weekly Epidemiological Update on COVID-19–27 July 2021* (WHO, 2021).

[2] Slaoui, M., Greene, S. E. & Woodcock, J. Bridging the gap at warp speed—delivering options for preventing and treating Covid-19. *N. Engl. J. Med*. 10.1056/NEJMp2028535 (2020). 10.1056/NEJMp2028535 32931679

[3] The WHO Rapid Evidence Appraisal for COVID-19 Therapies (REACT) Working Group. Association between administration of systemic corticosteroids and mortality among critically ill patients with COVID-19: a meta-analysis. *JAMA*10.1001/jama.2020.17023 (2020). 10.1001/jama.2020.17023 PMC7489434 32876694

[4] Beigel, J. H. et al. Remdesivir for the treatment of Covid-19—preliminary report. *N. Engl. J. Med*. 10.1056/NEJMoa2007764 (2020). 10.1056/NEJMc2022236 32649078

[5] Spinner CD (2020). Effect of remdesivir vs standard care on clinical status at 11 days in patients with moderate COVID-19: a randomized clinical trial. JAMA.

[6] Gautret P (2020). Hydroxychloroquine and azithromycin as a treatment of COVID-19: results of an open-label non-randomized clinical trial. Int. J. Antimicrob. Agents.

[7] Lagier J-C (2020). Outcomes of 3,737 COVID-19 patients treated with hydroxychloroquine/azithromycin and other regimens in Marseille, France. Travel Med. Infect. Dis..

[8] Yao X (2020). In vitro antiviral activity and projection of optimized dosing design of hydroxychloroquine for the treatment of severe acute respiratory syndrome coronavirus 2 (SARS-CoV-2). Clin. Infect. Dis..

[9] US Food and Drug Administration. Coronavirus (COVID-19) update: FDA reiterates importance of close patient supervision for ‘off-label’ use of antimalarial drugs to mitigate known risks, including heart rhythm problems. *FDA*. https://www.fda.gov/news-events/press-announcements/coronavirus-covid-19-update-fda-reiterates-importance-close-patient-supervision-label-use (2020).

[10] Chatre C, Roubille F, Vernhet H, Jorgensen C, Pers Y-M (2018). Cardiac complications attributed to chloroquine and hydroxychloroquine: a systematic review of the literature. Drug Saf..

[11] Ray WA, Murray KT, Hall K, Arbogast PG, Stein CM (2012). Azithromycin and the risk of cardiovascular death. N. Engl. J. Med..

[12] Borba MGS (2020). Effect of high vs low doses of chloroquine diphosphate as adjunctive therapy for patients hospitalized with severe acute respiratory syndrome coronavirus 2 (SARS-CoV-2) infection: a randomized clinical trial. JAMA Netw. Open.

[13] Mercuro NJ (2020). Risk of QT interval prolongation associated with use of hydroxychloroquine with or without concomitant azithromycin among hospitalized patients testing positive for coronavirus disease 2019 (COVID-19). JAMA Cardiol..

[14] Drew BJ (2010). Prevention of Torsade de Pointes in hospital settings. Circulation.

[15] Skipper CP (2020). Hydroxychloroquine in nonhospitalized adults with early COVID-19. Ann. Intern. Med..

[16] Mitjà, O. et al. Hydroxychloroquine for early treatment of adults with mild covid-19: a randomized-controlled trial. *Clin. Infect. Dis. Off. Publ. Infect. Dis. Soc. Am*. 10.1093/cid/ciaa1009 (2020). 10.1093/cid/ciaa1009 PMC7454406 32674126

[17] Johnston, C. et al. Hydroxychloroquine with or without azithromycin for treatment of early SARS-CoV-2 infection among high-risk outpatient adults: a randomized clinical trial. *EClinicalMedicine* **33**, 100773 (2021). 10.1016/j.eclinm.2021.100773 PMC7912360 33681731

[18] Inan OT (2020). Digitizing clinical trials. Npj Digit. Med..

[19] Boulware DR (2020). A randomized trial of hydroxychloroquine as postexposure prophylaxis for Covid-19. N. Engl. J. Med..

[20] Barnabas, R. V. et al. Hydroxychloroquine as postexposure prophylaxis to prevent severe acute respiratory syndrome coronavirus 2 infection. *Ann. Intern. Med*. 10.7326/M20-6519 (2020). 10.7326/M20-6519 PMC7732017 33284679

[21] Lenze EJ (2020). Fluvoxamine vs placebo and clinical deterioration in outpatients with symptomatic COVID-19: a randomized clinical trial. JAMA.

[22] Rajkomar A, Dean J, Kohane I (2019). Machine learning in medicine. N. Engl. J. Med..

[23] Krows, M. et al. Remote clinical trial methods result in recruitment from 44 states and high retention during pandemic. in *International AIDS Conference COVID-19 Prevention Conference* (2021).

[24] Tu Y-P (2020). Swabs collected by patients or health care workers for SARS-CoV-2 testing. N. Engl. J. Med..

[25] Perez MV (2019). Large-scale assessment of a smartwatch to identify atrial fibrillation. N. Engl. J. Med..

[26] Packer DL (2019). Effect of catheter ablation vs antiarrhythmic drug therapy on mortality, stroke, bleeding, and cardiac arrest among patients with atrial fibrillation: the CABANA randomized clinical trial. JAMA.

[27] Yap YG, Camm AJ (2003). Drug induced QT prolongation and torsades de pointes. Heart.

[28] Cheung CC, Davies B, Gibbs K, Laksman ZW, Krahn AD (2020). Multilead QT screening is necessary for QT measurement. JACC Clin. Electrophysiol..

[29] Giudicessi John, R. et al. Artificial intelligence-enabled assessment of the heart rate corrected QT interval using a mobile electrocardiogram device. *Circulation* **143**, 1274–1286 (2021). 10.1161/CIRCULATIONAHA.120.050231 33517677

[30] U. S. Food and Drug Administration. *510(k) Premarket Notification K183319: Triangle System* (FDA, 2019).

[31] Lieberman, J. A. et al. Comparison of commercially available and laboratory-developed assays for in vitro detection of SARS-CoV-2 in clinical laboratories. *J. Clin. Microbiol*. **58**, 10.1128/JCM.00821-20 (2020). 10.1128/JCM.00821-20 PMC7383518 32350048

[32] Powers JH (2016). Development of the Flu-PRO: a patient-reported outcome (PRO) instrument to evaluate symptoms of influenza. BMC Infect. Dis..

[33] Harris PA (2019). The REDCap consortium: building an international community of software platform partners. J. Biomed. Inform..

[34] AliveCor. Compatibility. *AliveCor Support* https://alivecor.zendesk.com/hc/en-us#compatibility Accessed - (December 2021).

[35] US Census Bureau. QuickFacts: United States. https://www.census.gov/quickfacts/fact/table/US/PST045219 Accessed - (December 2021).

[36] Wosik J (2020). Telehealth transformation: COVID-19 and the rise of virtual care. J. Am. Med. Inform. Assoc..

[37] Thokala P (2013). Telemonitoring after discharge from hospital with heart failure: cost-effectiveness modelling of alternative service designs. BMJ Open.

[38] Snoswell CL, Taylor ML, Caffery LJ (2019). The breakeven point for implementing telehealth. J. Telemed. Telecare.

[39] Hoffman, D. A. Increasing access to care: telehealth during COVID-19. *J. Law Biosci*. **7**, lsaa043 (2020). 10.1093/jlb/lsaa043 PMC7337821 32843985

[40] Food and Drug Administration, HHS. (2005). International Conference on Harmonisation; guidance on E14 clinical evaluation of QT/QTc interval prolongation and proarrhythmic potential for non-antiarrhythmic drugs; availability. Notice. Fed. Regist..

[41] Bouvy JC, Koopmanschap MA, Shah RR, Schellekens H (2012). The cost-effectiveness of drug regulation: the example of thorough QT/QTc studies. Clin. Pharmacol. Ther..

[42] Varma, N. et al. 2021 ISHNE/HRS/EHRA/APHRS Expert Collaborative Statement on mHealth in Arrhythmia Management: Digital Medical Tools for Heart Rhythm Professionals: From the International Society for Holter and Noninvasive Electrocardiology/Heart Rhythm Society/European Heart Rhythm Association/Asia-Pacific Heart Rhythm Society | Circulation: Arrhythmia and Electrophysiology. *Circ. Arrhythm. Electrophysiol.* **14**, 10.1161/CIRCEP.120.009204 (2021). 10.1161/CIRCEP.120.009204 PMC7892205 33573393

[43] Guanzon AV, Crouch MA (2004). Phase IV trial evaluating the effectiveness and safety of dofetilide. Ann. Pharmacother..

[44] Naksuk N (2019). Potentially modifiable factors of dofetilide-associated risk of torsades de pointes among hospitalized patients with atrial fibrillation. J. Interv. Card. Electrophysiol. Int. J. Arrhythm. Pacing.

[45] Goldstein EJC, Owens RC, Nolin TD (2006). Antimicrobial-associated QT interval prolongation: pointes of interest. Clin. Infect. Dis..

[46] Giudicessi John R, Noseworthy Peter A, Ackerman Michael J (2019). The QT interval. Circulation.

