# Supplementary material for: Implementation of a fully remote randomized clinical trial with cardiac monitoring
Source: Commun Med (Lond). 2021 Dec 20;1:62. doi: 10.1038/s43856-021-00052-w (PMC9053200; doi:10.1038/s43856-021-00052-w)
Supplement: Supplementary file 1 — Description of Additional Supplementary Files [file 43856_2021_52_MOESM1_ESM.pdf]

Description of Additional Supplementary Files

File Name: Supplementary Data 1

Description: This supplementary data file is presented as an excel file containing two tabs. The first tab includes the raw ECG adherence data and ECG parameters data. The second tab includes the daily adherence data for the different types of data collected - Survey, Swab and ECG
