# Supplementary material for: Implementation of a fully remote randomized clinical trial with cardiac monitoring
Source: Commun Med (Lond). 2021 Dec 20;1:62. doi: 10.1038/s43856-021-00052-w (PMC9053200; doi:10.1038/s43856-021-00052-w)
Supplement: Supplementary file 3 — Reporting Summary [file 43856_2021_52_MOESM3_ESM.pdf]

nature research

Corresponding author(s): Arun R Sridhar, MD, MPH

Last updated by author(s): Oct 24, 2021

Reporting Summary

Nature Research wishes to improve the reproducibility of the work that we publish. This form provides structure for consistency and transparency in reporting. For further information on Nature Research policies, see our Editorial Policies and the Editorial Policy Checklist.

Statistics

For all statistical analyses, confirm that the following items are present in the figure legend, table legend, main text, or Methods section.

n/a	Confirmed	
☐	☒	The exact sample size (*n*) for each experimental group/condition, given as a discrete number and unit of measurement
☐	☒	A statement on whether measurements were taken from distinct samples or whether the same sample was measured repeatedly
☒	☐	The statistical test(s) used AND whether they are one- or two-sided<br>*Only common tests should be described solely by name; describe more complex techniques in the Methods section.*
☒	☐	A description of all covariates tested
☒	☐	A description of any assumptions or corrections, such as tests of normality and adjustment for multiple comparisons
☐	☒	A full description of the statistical parameters including central tendency (e.g. means) or other basic estimates (e.g. regression coefficient) AND variation (e.g. standard deviation) or associated estimates of uncertainty (e.g. confidence intervals)
☒	☐	For null hypothesis testing, the test statistic (e.g. *F*, *t*, *r*) with confidence intervals, effect sizes, degrees of freedom and *P* value noted<br>*Give P values as exact values whenever suitable.*
☒	☐	For Bayesian analysis, information on the choice of priors and Markov chain Monte Carlo settings
☒	☐	For hierarchical and complex designs, identification of the appropriate level for tests and full reporting of outcomes
☒	☐	Estimates of effect sizes (e.g. Cohen's *d*, Pearson's *r*), indicating how they were calculated

Our web collection on statistics for biologists contains articles on many of the points above.

Software and code

Policy information about availability of computer code

Data collection	REDCap 11.1.2, proprietary AliveCor and Mayo Clinic software as described in the manuscript
Data analysis	STATA 17.0

For manuscripts utilizing custom algorithms or software that are central to the research but not yet described in published literature, software must be made available to editors and reviewers. We strongly encourage code deposition in a community repository (e.g. GitHub). See the Nature Research guidelines for submitting code & software for further information.

Data

Policy information about availability of data

All manuscripts must include a data availability statement. This statement should provide the following information, where applicable:

- Accession codes, unique identifiers, or web links for publicly available datasets
- A list of figures that have associated raw data
- A description of any restrictions on data availability

Source data is provided in Supplementary data 1. Remaining data is available from the corresponding author upon reasonable request

Field-specific reporting

Please select the one below that is the best fit for your research. If you are not sure, read the appropriate sections before making your selection.

☒ Life sciences ☐ Behavioural & social sciences ☐ Ecological, evolutionary & environmental sciences

For a reference copy of the document with all sections, see nature.com/documents/nr-reporting-summary-flat.pdf

Life sciences study design

All studies must disclose on these points even when the disclosure is negative.

Sample size	Sample size methods described previously, see: Johnston C, Brown ER, Stewart J, Karita HCS, Kissinger PJ, Dwyer J, Hosek S, Oyedele T, Paasche-Orlow MK, Paolino K, Heller KB, Leingang H, Haugen HS, Dong TQ, Bershteyn A, Sridhar AR, Poole J, Noseworthy PA, Ackerman MJ, Morrison S, Greninger AL, Huang ML, Jerome KR, Wener MH, Wald A, Schiffer JT, Celum C, Chu HY, Barnabas RV, Baeten JM; COVID-19 Early Treatment Study Team. Hydroxychloroquine with or without azithromycin for treatment of early SARS-CoV-2 infection among high-risk outpatient adults: A randomized clinical trial. EClinicalMedicine. 2021 Mar;33:100773. doi: 10.1016/j.eclinm.2021.100773. Epub 2021 Feb 27. PMID: 33681731; PMCID: PMC7912360.
Data exclusions	No data were excluded from analysis. Participants were excluded prior to data collection if they did not meet inclusion criteria or met any of the exclusion criteria.
Replication	Replication was not performed as this was a randomized clinical trial.
Randomization	Participants were randomized by household.
Blinding	This was a double-blind trial.

Reporting for specific materials, systems and methods

We require information from authors about some types of materials, experimental systems and methods used in many studies. Here, indicate whether each material, system or method listed is relevant to your study. If you are not sure if a list item applies to your research, read the appropriate section before selecting a response.

Materials & experimental systems

n/a	Involved in the study	
☒	☐	Antibodies
☒	☐	Eukaryotic cell lines
☒	☐	Palaeontology and archaeology
☒	☐	Animals and other organisms
☐	☒	Human research participants
☐	☒	Clinical data
☒	☐	Dual use research of concern

Methods

n/a	Involved in the study	
☒	☐	ChIP-seq
☒	☐	Flow cytometry
☒	☐	MRI-based neuroimaging

Human research participants

Policy information about studies involving human research participants

Population characteristics	A total of 231 participants were included from 205 households, of which, 218 initiated study medication and transmitted ECG data. The median age of participants was 37 (range 18-78) and 19 (8.7%) were 60 or older. Participants identifying as Hispanic or Latinx made up 29.8%. English was the preferred language of 90.8% and 9.1% preferred Spanish.
Recruitment	Nationwide social media advertising was employed for recruitment. Participants were screened via web interface, secure video conference, telephone, or text messaging. Due to the recruitment strategy which involved internet and social media campaign, our participants could be a self-selected population. This may result in bias toward higher adherence rate compared to true population performance.
Ethics oversight	The study was approved by the Western Institutional Review Board with reliance agreements from the collaborating institutions. An external and independent data and safety monitoring board provided oversight.

Note that full information on the approval of the study protocol must also be provided in the manuscript.

Clinical data

Policy information about clinical studies

All manuscripts should comply with the ICMJE guidelines for publication of clinical research and a completed CONSORT checklist must be included with all submissions.

Clinical trial registration	ClinicalTrials.gov - NCT04354428
Study protocol	Available from ClinicalTrials.gov - NCT04354428, in addition to the supplementary material.
Data collection	The trial was conducted entirely remotely, from enrollment through data collection and follow-up. Five U.S. institutions enrolled participants, including Ruth M. Rothstein CORE Center - Cook County Health (Chicago, Illinois, United States), Tulane University (New Orleans, Louisiana, United States), Boston University (Boston, Massachusetts, United States), SUNY Upstate Medical University (Syracuse, New York, United States), and the University of Washington (Seattle, Washington, United States).
Outcomes	Our manuscript is primarily methodological and describes qualitative and quantitative measures of adherence and success with digital clinical trials technologies, focusing on adherence to remote QT interval monitoring.
